# The Human Teredo

**Published:** 1913-04

**Authors:** 


### The Human Teredo.

The increase of nervousness and inefficiency seen on every hand is seldom ascribed to its real cause. So much has been said about public sanitation and personal hygiene that the most important function of the body, nerve and brain efficiency, has been neglected. Why was neurasthenia not known fifty years ago? Why have the diseases of the lungs and heart increased 150 per cent in thirty years? Why is it difficult to find men fully capable of filling places of great responsibility, or those requiring technical skill? The answer is because of a progressive loss of nerve-efficiency, as a result of our modern civilization. The spirit of keeping up appearances and the necessity of bending every nerve to meet competition in business, school, church, clubs and the home has placed such a stress on nerve function that it is losing its vigor and tonicity. If men of exceptional capacity feel this stress, what are the great mass of humanity with less endowment to do?

Dr. White's paper in this issue of The Journal calls attention to this lamentable condition and it would be well if some of us would heed his advice.

While the adjustment of social conditions is an evolutionary process, it is a part of that process for society to agitate and develop sentiment in the light of its knowledge. It is up to us of this generation to utilize the laws of eugenics. Indeed, applied heredity is the only means by which enlightened sentiment can cope with the situation and make the fruits of our improved environments of any consequence.

Then may it not be said that the "mad rush" of Americanism is a species of human teredo, burrowing to the very heart of our existence?

